# Farnesol Inhibits PI3 Kinase Signaling and Inflammatory Gene Expression in Primary Human Renal Epithelial Cells

**DOI:** 10.3390/biomedicines11123322

**Published:** 2023-12-15

**Authors:** Aline Müller, Maria Lozoya, Xiaoying Chen, Volkmar Weissig, Mahtab Nourbakhsh

**Affiliations:** 1 Department of Geriatric Medicine, RWTH Aachen University Hospital, 52074 Aachen, Germany; almueller@ukaachen.de (A.M.); xchen@ukaachen.de (X.C.); 2 College of Pharmacy, Midwestern University, Glendale, AZ 85308, USA; mlozoy@midwestern.edu (M.L.); vweiss@midwestern.edu (V.W.)

**Keywords:** human, renal proximal tubule epithelial cells, TNF-α, IL-1β, nanoliposomes, farnesol, PI3K

## Abstract

Chronic inflammation and elevated cytokine levels are closely associated with the progression of chronic kidney disease (CKD), which is responsible for the manifestation of numerous complications and mortality. In addition to conventional CKD therapies, the possibility of using natural compounds with anti-inflammatory potential has attracted widespread attention in scientific research. This study aimed to study the potential anti-inflammatory effects of a natural oil compound, farnesol, in primary human renal proximal tubule epithelial cell (RPTEC) culture. Farnesol was encapsulated in lipid-based small unilamellar vesicles (SUVs) to overcome its insolubility in cell culture medium. The cell attachment of empty vesicles (SUVs) and farnesol-loaded vesicles (farnesol-SUVs) was examined using BODIPY, a fluorescent dye with hydrophobic properties. Next, we used multiple protein, RNA, and protein phosphorylation arrays to investigate the impact of farnesol on inflammatory signaling in RPTECs. The results indicated that farnesol inhibits TNF-α/IL-1β-induced phosphorylation of the PI3 kinase p85 subunit and subsequent transcriptional activation of the inflammatory genes TNFRSF9, CD27, TNFRSF8, DR6, FAS, IL-7, and CCL2. Therefore, farnesol may be a promising natural compound for treating CKD.

## 1. Introduction

Chronic kidney disease (CKD) is characterized by the progressive loss of kidney function over time [1]. Chronic inflammation and excessive cytokine levels have been recognized as the key pathogenic mechanism for CKD in epidemiological studies [2]. In vitro studies of renal pathophysiology are especially challenging due to the complexity of nephrons, which are composed of various tubular segments and functionally different epithelial cells [3]. Renal proximal tubular epithelial cells (RPTECs) are highly sensitive to inflammatory cytokines, representing the primary target of inflammation in the renal system [4]. Accordingly, targeting the RPTEC cytokine response is considered a rational strategy in the management of CKD [2]. The culture of human primary RPTECs was established as a valuable tool for the in vitro study of renal pathologies related to multiple conditions, such as hypertension, diabetes, fibrosis, chronic inflammation, infection, and drug toxicity [2]. Through paracrine signaling, TNF-α induces interleukin (IL)-8 and E-selectin expression in different types of cells and enhances the expression levels and secretion of IL-1β by macrophages. In vitro, however, recombinant TNF-α and interleukin (IL)-1 proteins are usually combined (TNF-α/IL-1β) to induce a general inflammatory response in primary renal cells [5,6,7,8,9]. 

TNF-α/IL-1β-induced signaling pathways lead to the activation of a highly related set of transcription factors and genes that are involved in the innate immune response [10]. Phosphoinositide 3 kinase (PI3K) class IA plays a central role in signal transduction induced by various cytokines. PI3K is composed of two subunits: the regulatory p85 subunit and the p110 catalytic p110 subunit. Multiple phosphorylation sites in the regulatory subunit have been identified and implicated in the release and activation of the catalytic subunit p110. Upon activation, p110 can induce multiple downstream signaling cascades, including protein kinase B (Akt) and c (PKC), Ras, and NFκB [11,12]. Previous proteomic studies revealed that the majority of PI3K downstream signals were activated in TNF-α/IL-1β- stimulated renal cells [10]. 

Isoprenoids constitute a large and highly diverse family of natural compounds with important medical properties. The isoprenoid farnesol is a component of essential plant oils and an intermediate in the synthesis of cholesterol. The miscellaneous effects of farnesol have been primarily reported in animal studies. The topical or oral administration of farnesol as a dietary supplement was reported to exert beneficial effects on microbial infection, tumor growth, oxidative stress, and inflammation in rodent models [13,14,15]. Moreover, farnesol produced by *candida albicans* was previously reported to induce the expression of inflammatory cytokines in a transformed murine macrophage line [16]. However, the strong hydrophobicity of farnesol has limited a more detailed examination of its molecular effects in cell culture. 

Nanoliposomes are the most frequently used nanocarriers for the application of active hydrophobic and hydrophilic molecules in vitro and in vivo [17]. They are highly biocompatible and can enhance drug solubility, release, and target specificity under experimental and clinical conditions [17]. Several drugs or antigens have been successfully encapsulated in liposomes and utilized for the immunization and treatment against cancer and infections [17]. Another advantage of liposomes is that their composition and structure can be modified to match the compound, target cell, organ, or medical application. Recently, we encapsulated farnesol in small unilamellar vesicles (SUVs) consisting of the phospholipid L-α-phosphatidylcholine (PC) and the lipid 1,2-dioleoyl-3-trimethylammonium-propane (DOTAP). These SUVs have been successfully applied to human skeletal myoblasts [18]. The aim of the current study was to examine the effect of farnesol-SUVs on the inflammatory response of primary human RPTECs and to identify the target genes and signaling factors of farnesol that may be relevant to renal inflammation and CKD. 

## 2. Materials and Methods

### 2.1. Nanoliposome Preparation and Quantification

PC and DOTAP (Avanti Polar Lipids Inc., Alabaster, AL, USA) were utilized in 98:2% molar ratios to prepare liposomes with or without adding 4 mM farnesol (Sigma-Aldrich, Inc., St. Louis, MO, USA) using the lipid film hydration method [18]. All components were solved in chloroform and mixed thoroughly for 5 min. A rotary vacuum evaporator (Yamato, RE-46, Santa Clara, CA, USA) was used to remove the chloroform and form a thin lipid film which was then suspended in a final lipid concentration of 20 mg/mL in 5 mM HEPES solution (4-(-2-hydroxyethyl) piperizazine-1-ethanesulfonic acid) pH 7.4. The resulting liposomal suspension was sonicated (Sonic Dismembrator Model 100, 1β (Fisher Scientific, Pittsburgh, PA, USA) at 5 W for 45 min in an ice bath to obtain the SUVs. Titanium particles resulting from sonication were removed by centrifugation at 1550× *g* (RCF) for 15 min at 4 °C. The encapsulation efficiency of farnesol was 98.15% as determined by reverse-phase high-performance liquid chromatography (HPLC) analysis, as described previously [18]. A Nano ZS Zetasizer (Malvern Panalytical, Westborough, MA, USA) was used to measure the hydrodynamic diameters of the liposomes in triplicate via dynamic light scattering (DLS) at 25 °C. The results were reported as size by volume (nm) and polydispersity index in this study. Before application to cells, liposome suspensions were sterile-filtered using disposable filters with a pore size of 0.22 µm. 

### 2.2. Cell Culture

Primary human RPTECs (CC-2553) were obtained from Lonza and tested positive for gamma-GTP and pancytokeratin and negative for mycoplasma, bacteria, yeast, fungi, HIV, hepatitis B, and C. (Basel, Switzerland). RPTECs were maintained in Renal Epithelial Cell Growth Medium (REGM) with BulletKit supplements (CC-3190, Lonza, Basel, Switzerland) at 37 °C and 5% CO_2_, according to the manufacturer’s instructions. For all experiments, RPTECs at the 7th doubling cycle were seeded at a density of 20,000 or 45,000 cells/cm^2^ for protein and phosphorylation or mRNA experiments. Cells were left untreated or treated with REGM with BulletKit supplements (CC-3190, Lonza) treated with 1:200 of 4 mM farnesol, SUV, or farnesol-SUV for at least 48 h. RPTECs were then left untreated or treated with 10 ng/mL purified recombinant TNF-α and 0.25 ng/mL IL-1β (Thermo Fisher Scientific, Waltham, MA, USA) for 0.5, 6 or 24 h before being harvested for phosphorylation, mRNA, or protein analyses, respectively.

### 2.3. Lipid Vesicle Imaging

RPTECs (25,000 cells/cm^2^) were treated as described in the Results section (Section 3.2) and carefully washed in PBS (P04-36500, PAN Biotech, Aidenbach, Germany). Cells were incubated with 1 µM lipophilic green fluorescent BODIPY 493/503 (D3922, Thermo Fisher Scientific) in serum-free Renal Epithelial Basal Medium (REBM) (CC-3191, Lonza) for 15 min at 37 °C and 5% CO_2_. After staining, BODIPY was carefully removed by washing the cells with PBS before imaging.

### 2.4. Monitoring Mitochondrial Abundance and Function

RPTECs (25,000 cells/cm^2^) were treated as described in Section 3.2 and carefully washed in PBS (P04-36500, PAN Biotech, Aidenbach, Germany). Cells were incubated with 200 nM MitoTracker Red CMXRos, a derivative of red fluorescent X-rosamine (M7512, Thermo Fisher Scientific, Waltham, MA, USA), or with 1 µM of a chemically reduced form of tetramethylrosamine, MitoTracker Orange CM-H2TMRos (M7511, Thermo Fisher Scientific) in serum-free REBM for 60 or 30 min, respectively. Cells were washed in serum-free REBM before imaging.

### 2.5. Fluorescence Microscopy/Imaging

Cell images were captured using an automated inverted microscope (DMI4000B, Leica Microsystems, Wetzlar, Germany) and 450–490 nm (I3) after BODIPY staining or a 515–560 nm filter (the N2.1) after mitochondria staining. Cell images were processed with Diskus Software v5.0 (Hilgers Technisches Buero e.K., Königswinter, Germany).

### 2.6. Multiplex Protein Quantification

The expression of BDNF, CCL2, CD27, CD40, CXCL8, DR6, EGF, Eotaxin (CCL11), FAS, FGF-basic, G-CSF/CSF-3, GITR, GM-CSF, GRO alpha (CXCL1), HGF, IFN alpha, IFN gamma, IL-1 alpha, IL-1 beta, IL-10, IL-12p70, IL-13, IL-15, IL-17A (CTLA-8), IL-18, IL-1RA, IL-2, IL-21, IL-22, IL-23, IL-27, IL-2R, IL-31, IL-4, IL-5, IL-6, IL-7, IL-9, IP-10, (CXCL10), LIF, MIG, MIP-1 alpha (CCL3), MIP-1 beta (CCL4), RANTES (CCL5), SDF-1 alpha, TNF alpha, TNF beta, TNF-RI, TNF-RII, TNFRSF14, TNFRSF8, TNFRSF9, TRAIL-R1, TRAIL-R2, and VEGF-A was measured using Procarta Plex Human Cytokine/Chemokine Panels (EPX340-12167-901, EPX120-15802-901, EPX080-12186-901, EPX-10-MXEPUYZ) from Thermo Fisher Scientific. The manufacturer’s instructions were rigorously followed to prepare RPTEC protein extracts in Procarta Plex Cell Lysis Buffer (EPX-99999-000, Thermo Fisher Scientific) and to analyze the samples using Luminex xMAP technology-based Magpix (Thermo Fisher Scientific). Pierce 660 nm Protein Assay Kit (22662, Thermo Fisher Scientific) was used to determine the total protein concentration in cell extracts according to the manufacturer’s instructions.

### 2.7. Multiplex mRNA Quantification

The RNA extracts from RPTECs were quantified using preconfigured Quantigene Plex assays from Thermo Fisher Scientific based on direct hybridization to specifically designed capture extenders (CE), label extenders (LE), and blocking probes (BL). Sequences were recorded in the NCBI nucleotide database (https://www.ncbi.nlm.nih.gov/nucleotide/, accessed 8 December 2023) with the accession numbers NM_001413263 (CD27), NM_000043 (FAS), NM_000880 (IL7), NM_014452 (DR6), NM_002982 (CCL2), NM_001243 (TNFRSF8), NM_001561 (TNFRSF9), NM_002046 (GAPDH), and NM_000194 (HPRT1). The manufacturer’s instructions (Thermo Fisher Scientific) were strictly followed to prepare RPTEC RNA extracts in QG Sample Processing Kit (QS0100, Thermo Fisher Scientific). Luminex xMAP technology-based Magpix (Thermo Fisher Scientific) was utilized to analyze the samples and the levels of RNA expression were normalized to the geometric mean of GAPDH and HPRT1 RNA expression levels, according to the manufacturer’s instructions. 

### 2.8. Protein Phosphorylation Array

NF-KB Signaling Phospho Antibody Array (PNK 215) and TGF-beta Signaling Phospho Antibody Array (PTG 176) from Full Moon Biosystems (Sunnyvale, CA, USA) are glass-based antibody arrays for broad-scope protein phosphorylation profiling and screening in cells. A total of 215 highly specific antibodies related to the NF-κB pathway and 176 antibodies linked to the TGF-beta signaling pathway in RPTEC were used. Following the indicated treatments, the supernatant was removed, and RPTECs were washed in cold PBS three times. According to the manufacturer’s instructions for the Antibody Array Assay Kit (KAS02, Full Moon Biosystems, Sunnyvale, CA, USA), cells were solubilized in Extraction Buffer (included in Array Assay Kit) with protease and phosphatase inhibitors (ab201119, Abcam, Cambridge, UK). Pierce 660 nm Protein Assay Kit (22662, Thermo Fisher Scientific) was used to determine the total protein concentration in cell extracts according to the manufacturer’s instructions. Arrays were incubated with 63 µg of cellular proteins at room temperature for 2 h. Then, arrays were washed and scanned using a fluorescence scanner (Full Moon BioSystems). Array images were analyzed using ImageJ software v1.54g (https://imagej.nih.gov/ij/, accessed on 15 November 2023).

### 2.9. Statistical Analysis

All results are reported as the mean ± standard deviation (SD). Statistical analyses were performed using GraphPad Prism (v8, GraphPad Software, San Diego, CA, USA). One-way ANOVA with Tukey–Kramer multiple comparison tests for normally distributed or Kruskal–Wallis tests with Dunn’s multiple comparison tests for non-normally distributed data were used to test for statistical significance in the protein and RNA assays. For phosphorylation analysis, the statistical significance was tested using a one-sample *t* test for normally distributed or Wilcoxon signed-rank tests for non-normally distributed data. Differences with *p* values ≤ 0.01 were considered statistically significant.

## 3. Results

### 3.1. The Physicochemical Characteristics of SUVs and Farnesol-SUVs

In a previous study in human skeletal myoblasts, we previously established a procedure for the encapsulation of farnesol in SUVs composed of DOTAP and PC/soy [18]. For the current study, we followed the same formula and created two batches of SUVs, one with farnesol (farnesol-SUV) and one without farnesol (SUV). The empty SUVs were intended to serve as a control to exclude possible unintended effects of DOTAP and PC/soy in cells. Moreover, the size and polydispersity indexes (PDI) of farnesol-SUVs and SUVs were compared using DLS. We obtained comparable distributions of particle size by volume (Table 1). The PDIs of farnesol-SUV and SUV differed by 0.057, which is negligible according to the DSL standards [19]. The efficiency of farnesol encapsulation was 3.926 mM (98.15%), determined by reverse-phase high-performance liquid chromatography (HPLC), as described previously [18].

### 3.2. Uptake of SUVs and Farnesol-SUVs by RPTECs

**Figure 1:**
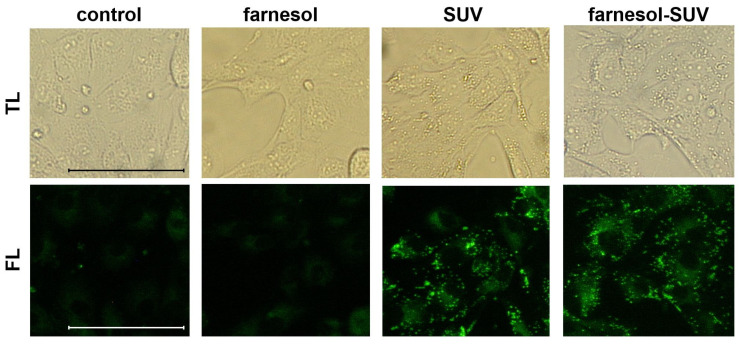
Equal uptake of SUVs and farnesol-SUVs by RPTECs. RPTECs were either not treated (control) or were treated with farnesol, SUVs, or farnesol-SUVs as indicated on the top of each panel. RPTECs were stained using BODIPY. All transmitted light (TL, **upper panel**) or fluorescence (FL, **lower panel**) images were captured using the same magnification and exposure times. Scale bars indicate 50 μm.

SUVs and farnesol-SUVs were shown to attach to human myoblasts [18]. However, the interaction of liposomes with cells can be influenced by the distinctive composition of the cell membrane [16,17]. Therefore, we examined the attachment of SUVs and farnesol-SUVs to RPTECs using the lipophilic fluorescent dye BODIPY. The cytoplasm of untreated RPTECs showed weak fluorescent signals due to the low content of intracellular lipids (Figure 1). Indistinguishable signals were obtained when RPTECs were treated with free farnesol, which is not soluble in cell culture medium. In contrast, SUV- or farnesol-SUV-treated RPTECs exhibited comparable and protuberant fluorescent signals. This finding indicated that SUVs and farnesol-SUVs were equally attached to RPTECs.

### 3.3. SUVs and Farnesol-SUVs Do Not Affect Mitochondrial Abundance or Activity in RPTECs

**Figure 2:**
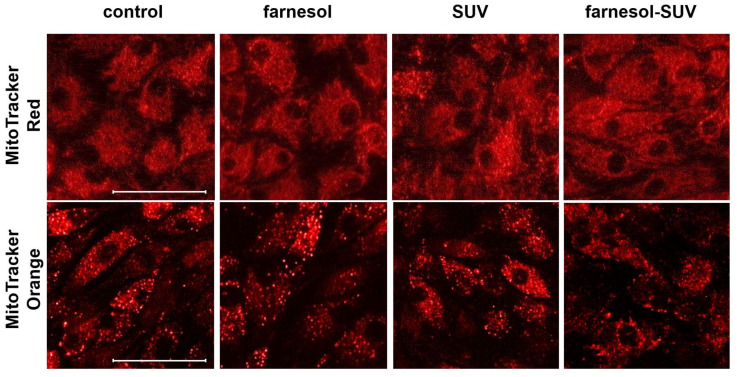
Abundance and oxidative activity of mitochondria in RPTECs. RPTECs were left untreated (control) or treated with SUVs or farnesol-SUVs, as indicated at the top of each panel. Mitochondria were stained using MitoTracker Red (**upper panel**) or MitoTracker Orange (**lower panel**). The presented images were captured at the same settings and exposure times and are representative of three independent sets of experiments. Scale bars indicate 50 μm.

The intracellular accumulation of some lipid species can cause an imbalance between the uptake and oxidation of fatty acids and interfere with mitochondrial activity, resulting in oxidative stress in renal cells [20,21]. Since SUVs and farnesol-SUVs are composed of lipid and phospholipids components, we used two different cell-permeable fluorescent probes to detect the mitochondrial abundance (MitoTracker Red) or oxidative activity (MitoTracker Orange) and to rule out any unintended effects on the mitochondrial activity in RPTECs. We found no significant differences between fluorescence signals obtained from untreated RPTECs and those treated with farnesol, SUVs, or farnesol, SUVs (Figure 2). Thus, SUVs and farnesol-SUVs do not affect mitochondrial abundance or activity in RPTECs. 

### 3.4. Farnesol-SUVs Inhibit TNF-α/IL-1β-Induced Expression of Inflammatory Proteins in RPTECs

**Figure 3:**
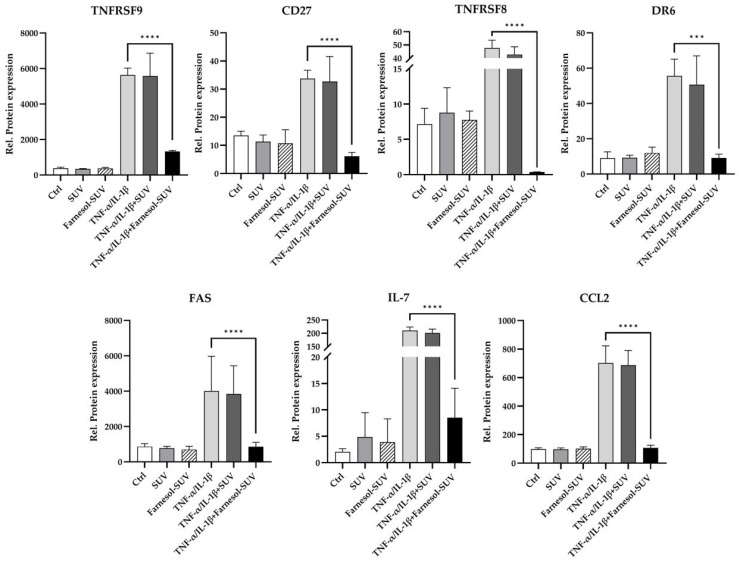
Farnesol-SUVs inhibit the TNF-α/IL-1β-induced expression of inflammatory proteins. RPTECs were left untreated or treated with SUV or farnesol-SUV for at least 48 h and then left unstimulated or stimulated with TNF-α /IL-1β for another 24 h. Cell extracts were analyzed for 55 chemokines/cytokines in triplicate. The relative protein expression was obtained through the normalization of the level of each protein to the total cell extract in each experiment (y-axis). The expression levels of seven proteins, TNFRSF9, CD27, TNFRSF8, DR6, FAS, IL-7, and CCL2, were inhibited by farnesol-SUV in RPTECS. The results are presented as the mean ± SD of the relative expression of each protein designated at the top of each diagram. Statistical significance was calculated using one-way ANOVA with the Tukey-Kramer multiple comparison test (TNFRSF9, CD27, FAS, IL-7, and CCL2) or Kruskal-Wallis test with Dunn’s multiple comparison test (TNFRSF8 and DR6) where appropriate. For the sake of simplicity, only the *p* values of significance for differences between TNF-α /IL-1β-stimulated cells, which were treated with farnesol-SUV or left untreated, are shown (asterisks). The differences between unstimulated (Ctrl, SUV, Farnesol-SUV) and respective TNF-α/IL-1β-stimulated cells (TNF-α/IL-1β, TNF-α/IL-1β+SUV, TNF-α/IL-1β+Farnesol-SUV) were <0.05. The differences between unstimulated cells (Ctrl, SUV, Farnesol-SUV) and between untreated and SUV-treated TNF-α/IL-1β-stimulated cells (TNF-α/IL-1β, TNF-α/IL-1β+SUV) were insignificant. *p* ≤ 0.001 (***). *p* ≤ 0.0001 (****).

The uptake of SUVs by RPTECs is a slower process than the activation of the inflammatory response to TNF-α/IL-1β stimulation. To achieve sufficient uptake of farnesol before TNF-α/IL-1β stimulation, RPTECs were pretreated with SUVs or farnesol-SUVs for at least 48 h or left untreated as a control. Next, RPTECs were stimulated with TNF-α/IL-1β or left untreated for another 24 h. All experiments were performed in triplicate to validate the outcomes. Next, we used preconfigured multiplex panels for the simultaneous detection of 55 chemokines/cytokines in all samples. Each sample was analyzed twice to obtain mean values and to ensure data accuracy. The amounts of chemokines/cytokines were normalized to the total amounts of cellular proteins in each sample to obtain relative protein expression (Figure 3, *y*-axis). Forty chemokines/cytokines were expressed at detectable levels (>1 fg/µg cell extract), including 26 chemokines/cytokines, which were significantly induced by TNF-α/IL-1β (Supplementary Table S1). This expression pattern highlighted the broad inflammatory response of RPTECs. In general, untreated control and SUV-treated RPTECs revealed indistinguishable results (Figure 3). Pretreatment with SUVs showed no effect on the stimulated or unstimulated expression of these proteins in RPTECs. Most importantly, farnesol-SUVs specifically affected the TNF-α/IL-1β-stimulated expression of seven inflammatory proteins, TNFRSF9, CD27, TNFRSF8, DR6, FAS, IL-7, and CCL2 (Figure 3). Thus, we suggested that farnesol can specifically downregulate the TNF-α/IL-1β-mediated expression of TNFRSF9, CD27, TNFRSF8, DR6, FAS, IL-7, and CCL2.

### 3.5. Farnesol-SUVs Inhibits TNF-α/IL-1β-Induced mRNA Expression of Inflammatory Genes in RPTECs

**Figure 4:**
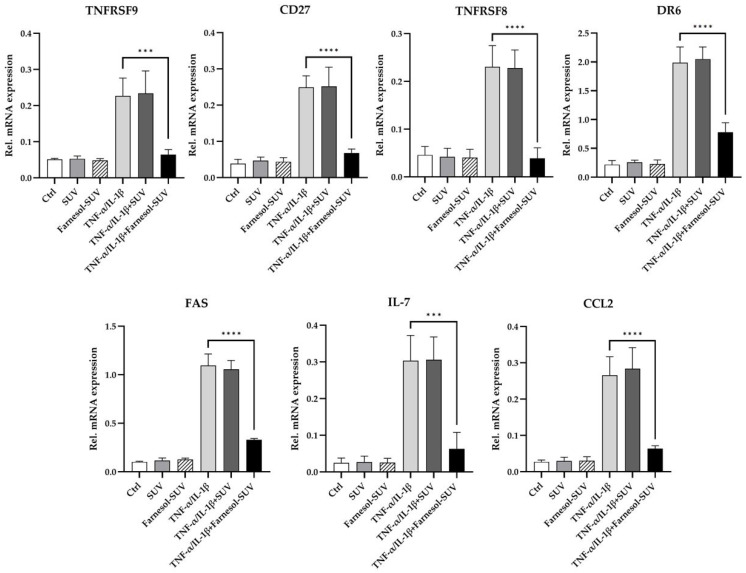
Farnesol inhibits TNF-α/IL-1β-mediated upregulation of inflammatory genes. RPTECs were left untreated or treated with SUV or farnesol-SUV for at least 48 h and then left unstimulated or stimulated with TNF-α/IL-1β for another 6 h. RPTECs were harvested, and total RNA was analyzed in triplicate using multiplex mRNA quantification to determine the mRNA expression levels of TNFRSF9, CD27, TNFRSF8, DR6, FAS, IL-7, and CCL2 that were normalized to the mRNA levels of GAPDH and HPRT1. The results are presented as the mean ± SD of the relative expression of mRNAs as indicated at the top of each diagram in three independent experiments. Statistical significance was calculated using one-way ANOVA with the Tukey-Kramer multiple comparison test. For the sake of simplicity, only the *p* value of significance for differences between TNF-α /IL-1β-stimulated cells which were treated with farnesol-SUV or left untreated are shown (asterisks). The differences between unstimulated (Ctrl, SUV, Farnesol-SUV) and respective TNF-α/IL-1β-stimulated cells (TNF-α/IL-1β, TNF-α/IL-1β+SUV, TNF-α/IL-1β+Farnesol-SUV) were <0.001. The differences between unstimulated cells (Ctrl, SUV, Farnesol-SUV) and between untreated and SUV-treated TNF-α/IL-1β-stimulated cells (TNF-α/IL-1β, TNF-α/IL-1β+SUV) were insignificant. *p* ≤ 0.001 (***). *p* ≤ 0.0001 (****).

To determine whether farnesol modulates the transcript levels of the seven downregulated proteins, we analyzed the mRNA expression levels of TNFRSF9, CD27, TNFRSF8, DR6, FAS, IL-7, and CCL2 using a multiplex hybridization assay. RPTECs were treated as described above in three independent experiments (Section 3.4). To obtain relative mRNA expression, the mRNA levels of the target genes were normalized to those of two housekeeping genes (Figure 4). The results were entirely correlated with the reported effects of farnesol on protein expression. Untreated control and SUV-treated RPTECs revealed indistinguishable mRNA expression patterns (Figure 4). Again, this ruled out any possible effects by empty SUVs. In contrast, farnesol-SUVs significantly reduced the levels of TNFRSF9, CD27, TNFRSF8, DR6, FAS, IL-7, and CCL2 mRNA expression in TNF-α/IL-1β-stimulated RPTECs specifically (Figure 4). These results strongly suggested that farnesol affects the TNF-α/IL-1β-stimulated signal transduction pathways that lead to the elevated gene expression of TNFRSF9, CD27, TNFRSF8, DR6, FAS, IL-7, and CCL2 genes.

### 3.6. Farnesol-SUVs Inhibits the Phosphorylation of Signaling Proteins in RPTECs

Members of the NFκB and TGF-β signaling pathways have been implicated in the TNF-α/IL-1β-stimulated activation of inflammatory genes, prompting us to identify those members that were potentially affected by farnesol-SUVs in TNF-α/IL-1β-stimulated RPTECs. We used preconfigured ELISA-based glass arrays that contained six replicates of 122 different antibodies against 82 differentially phosphorylated and unphosphorylated human signaling proteins (Supplementary Table S2). Due to the high cost of phosphorylation arrays, only two samples were considered for further studies, TNF-α/IL-1β-stimulated RPTECs pretreated with farnesol-SUV or left untreated. SUV control exhibited no effects on unstimulated or TNF-α/IL-1β-stimulated RPTECs and was rather dispensable for proposing a potential mechanism for farnesol-SUV.

**Figure 5:**
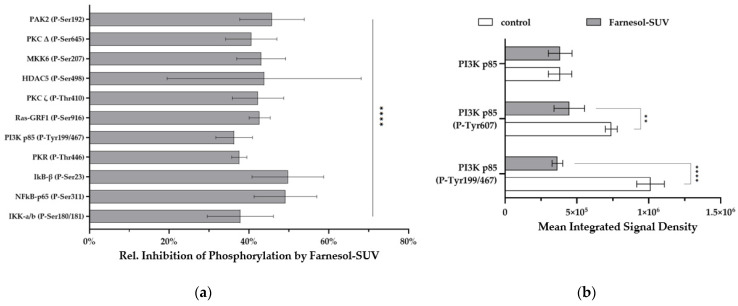
Farnesol inhibits the TNF-α/IL-1β-induced phosphorylation of signaling proteins. (**a**) RPTECs were left untreated or treated with farnesol-SUV and then stimulated with TNF-α/IL-1β for 0.5 h. The level of phosphorylated proteins was analyzed using preconfigured arrays of specific antibodies, each mounted as six replicates in equal amounts. The signals from SUV-farnesol-treated RPTECs were compared to the signals from control cells to obtain the relative inhibition of protein phosphorylation (y-axis) by farnesol-SUVs. Data are presented as the mean ± SD of six replicates. Statistical significance was calculated using one-sample *t* test (IκB-β (P-Ser23), PKR (P-Thr466), PI3K p85 (P-Tyr199/467), RAS-GRF1 (P-Ser916), PKCζ (P-Thr410), MKK6 (P-Serr207)) or Wilcoxon signed rank test (PAK2 (P-Ser192), PKCΔ (P-Ser645), HDAC5 (P-Ser207), NFκB-p65 (P-Ser311), IKK-a/b (P-Ser180/181)), as appropriate. (**b**) The diagram shows the mean integrated signal density of the PI3K p85 subunit or its phosphorylated isoforms, PI3K p85 (P-Tyr607) and PI3K p85 (P-Tyr199/467) (y-axis), in TNF-α/IL-1β-stimulated RPTECs that were left untreated (white bars) or treated with farnesol-SUVs (gray bars) before. Statistical significance was calculated using an unpaired *t* test or Mann-Whitney test, as appropriate. *p* ≤ 0.05 (**). *p* ≤ 0.0001 (****).

Previous studies have shown that the TNF-α/IL-1β stimulation of inflammatory pathways occurs within 15–45 min [22,23,24]. RPTECs were first pretreated with farnesol-SUVs or left untreated and then stimulated with TNF-α/IL-1β for 30 min before being subjected to protein extraction. Cell extracts were each incubated with two preconfigured arrays. After scanning, raw signal densities were extracted from array images and normalized to the background. We compared the phosphorylation signals from farnesol-SUV-treated RPTECs to those of control cells to provide a relative mean phosphorylation inhibition (Figure 5a). The phosphorylation of eleven proteins was significantly inhibited by farnesol-SUVs to lower than 50% (*p* ≤ 0.001, Figure 5a). Among them, farnesol-SUV inhibited p85 phosphorylation (Tyr467/Tyr199) to the lowest level in our experiments. PI3K p85 acts at a superior signaling position upstream of protein kinase C (PKC), Ras, IkκB kinase (IKK), or histone deacetylase 5 (HDAC5). Thus, PI3K p85 may be the key target of farnesol in RPTECs. Next, the precise comparison of the phosphorylated and unphosphorylated p85 isoforms in control and farnesol-SUV-treated cells emphasized the importance of Tyr467/Tyr199 phosphorylation in farnesol-mediated effects (Figure 5b). We compared the mean signal intensities from specific antibodies against unphosphorylated p85 and its phosphorylated isoforms at Tyr residues 607 and 199 or 467. Tyr (199/467) phosphorylation prompts the immediate release of catalytic subunit p110, whereas Tyr 607 regulates the later dimerization of p85 [23]. This comparison strengthens the initial hypothesis that farnesol acts at the early stage of cytokine-induced signaling in RPTECs. 

## 4. Discussion

Farnesol as a dietary supplement has been reported to have anti-allergic, anti-inflammatory, and anti-fibrotic effects through uncharacterized mechanisms. The current study utilized a liposome-based strategy to characterize the direct effects of farnesol on inflammatory signaling in RPTECs, which have long been recognized as in vitro models for the early stage testing of new compounds for kidney disorders [2]. We further demonstrated that farnesol interfered with the TNF-α/IL-1β-induced phosphorylation of the PI3K p85 subunit, its downstream signaling proteins, and the expression of a distinct set of genes involved in the renal inflammatory response.

Signaling pathway dynamics are primarily determined by cell phenotype and stimuli [25]. TNF-α and IL-1β are small peptides (17.3, 17.5 kDa) that are highly soluble in cell culture medium, granting the rapid activation of signaling pathways by binding to their specific receptors. RPTECs are the first point of contact for pathogens and cytokines in the kidney and thus rapidly respond to inflammatory signals. In the current study, we identified the significant activation of signaling proteins 30 min after TNF-α/IL-1β stimulation. This is in line with the previously described dynamics of signal transduction and accumulation of target mRNAs and proteins in primary human RPTECs [26,27,28]. This fast response to TNF-α/IL-1β rationalized the pretreatment of RPTECs with farnesol-SUVs in our study. 

The main advantage of using predesigned arrays was the ability to simultaneously examine a comprehensive set of potential farnesol cellular targets. We chose protein, RNA, and phospho-antibody arrays that were relevant to inflammation. However, some of the analytes are implicated in the regulation of cell growth and metabolism as well. The complete dataset revealed additional targets that may be potentially affected by farnesol to a lower or insignificant extent (Supplementary Tables S1 and S2). Because array experiments reflect a snapshot of events, it is conceivable that some of the observed minor effects here may become more substantial over an extended course of time. In fact, animal studies have reported diverse long-term effects of farnesol on metabolism and proliferation [13,29]. Nevertheless, the diversity of farnesol effects may be partially explained by the heterogeneous set of target genes depending on cell type or stimuli. In TNF-α/IL-1β-stimulated RPTECs, prior treatment with farnesol-SUV inhibited seven target genes, TNFRSF9, CD27, TNFRSF8, DR6, FAS, IL-7, and CCL2 (Figure 4). In a previous study of fatty acid-stimulated primary human skeletal myoblasts, pretreatment with farnesol-SUV repressed the expression of IL6 and CXCL8 [18]. In the current study, TNF-α/IL-1β-stimulated the expression of IL6, and CXCL8 was not affected by farnesol-SUV in RPTECs (Supplementary Table S1). In primary human skeletal myoblasts, 48 h of treatment with 0.5 µM farnesol in DMSO elevated the level of peroxisome proliferator-activated receptor-γ coactivator-1α (PGC-1α) by 1.7-fold [29]. Although this slight increase may be due to less efficient delivery of farnesol, its long-term effects may be different than its short-term effects. Nonetheless, a general limitation to ours and other studies is that the effective uptake of farnesol in cells is difficult to assess. Liposomes can deliver a significantly higher concentration of farnesol (3.926 mM) than DMSO. Moreover, we were able to track the attachment of liposomes to cells, while the delivery of farnesol by DMSO could not be monitored. The tracking of liposomes was especially relevant to our study because we compared the effects mediated by SUVs and farnesol-SUV in parallel experiments.

**Figure 6:**
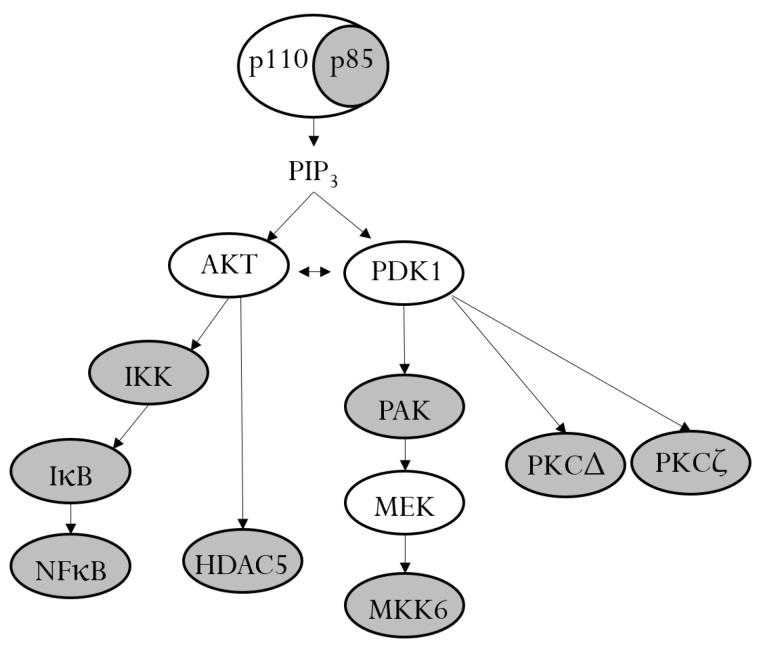
PI3K and its downstream signaling proteins. In unstimulated cells, the p110 catalytic subunit of PI3K is stabilized by dimerization with the regulatory p85 subunit. Upon activation, the p85 subunit becomes phosphorylated and releases p110, which leads to the production of PIP3 and the activation of the signaling kinases AKT and PDK1. AKT and PDK1 induce a cascade of downstream phosphorylation. Farnesol inhibits the phosphorylation of different members of the PI3K signaling pathway (gray ovals).

The analysis of phosphorylated proteins in TNF-α/IL-1β-stimulated RPTECs identified eleven target phosphoproteins of farnesol, including the PI3K p85 subunit and eight of its downstream signaling proteins, as schematically presented in Figure 6. This finding implies a key role of PI3K signaling in the regulation of the inflammatory response in RPTECs. Interestingly, recent studies have suggested an important role of the PI3K signaling pathway in renal dysfunction associated with diabetes or cardiorenal syndrome [30,31,32]. Although none of these studies were performed in RPTECs, it is tempting to speculate that farnesol may have a beneficial effect on renal malfunctions, which are associated with the dysregulation of PI3K signaling. 

The use of phosphorylation-detecting antibody arrays is associated with some limitations that are important to consider. First, the intensity of signals strongly depends on the quality and specificity of the detecting antibodies. Thus, phosphorylation signals obtained from different antibodies cannot be directly compared, while a direct comparison of the same antibody under different conditions is validated by replicates. For instance, the signal of phosphorylated p85 Tyr199/467 is not directly comparable to that of phosphorylated p85 Tyr607 (Figure 5b). Second, the data reflect the effects of farnesol on phosphorylation events within 30 min of TNF-α/IL-1β stimulation. Therefore, it is rather difficult to capture fast reversible phosphorylation, such AKT Ser473 or Thr308 [33]. Moreover, dephosphorylation at Thr308 and Ser473 was reported to promote AKT degradation [33]. Indeed, total AKT protein level in RPTECs pretreated with farnesol-SUV was reduced by 40% (AKT (Ab-326) and (Ab-474), Supplementary Table S2). Hence, the level of inhibition by farnesol on phosphorylation may change over the course of stimulation. This inhibition of farnesol target proteins involved in diverse cellular functions may expand its therapeutic applications.

The marked effects of farnesol on p85 phosphorylation implicate PI3K signaling pathway in the regulation of TNFRSF9, CD27, TNFRSF8, DR6, FAS, IL-7, and CCL2 genes. Indeed, the PI3K-regulated activation of TNFRSF8 and TNFRSF9 expression has been reported previously [34,35]. In vitro studies on the regulatory 5′-upstream gene sequences have suggested a possible link between PI3K signaling pathway and other farnesol target genes. For instance, CCL2 promoter was shown to comprise a functional binding site for NFκB p65, which activates CCL2 gene transcription in response to IL-1 and TNF-α [36]. Similarly, NFκB was reported to mediate the transcriptional activation of FAS gene in a rodent model [37]. The 5′ upstream regions of IL-7 and DR6 genes are associated with CpG islands, lacking classic transcriptional regulatory sequence elements, such as TATA or CAAT [38]. Although the TNF-α/IL-1β activated expression of IL-7 gene has been reported previously [39], the complexity of IL-7 and DR6 promoter regions has limited the further investigation. Thus, the direct involvement of PI3K signaling pathway in IL-7 and DR6 gene regulations remain to be addressed.

All farnesol target genes identified in our study were linked to CKD development and progression, which have been associated with chronic inflammation, oxidative stress, and ischemia [40]. TNFRSF9 and FAS receptor were identified as biomarkers of impaired kidney function and CKD in epidemiological studies [41,42]. Similarly, serum CD27 levels were strongly associated with kidney dysfunction and a low glomerular filtration rate in type 1 diabetes [43]. Moreover, TNFRSF8 and DR6 were previously linked to renal injury and ischemia [6,44]. Independent of the source of renal injury, tubular fibrosis seems to be a common mechanism of CKD progression and end-stage renal disease (ESRD) [45,46]. Interestingly, farnesol inhibited CCL2, which is also known as monocyte chemoattractant protein-1 (MCP-1), the most prominent chemokine-promoting tissue fibrosis [47]. The therapeutic inhibitors of renal fibrosis were shown to downregulate the expression of CCL2 in renal cells [48,49]. These observations are in line with our findings and may be the mechanism by which farnesol protects against renal inflammation and fibrosis. The role of IL-7 in this context remains elusive. A previous study on RPTECs from patients with IgA nephropathy reported that upregulated IL-7 expression attenuated cellular fibrosis induced by transforming growth factor β l [50]. In our study, we used RPTECs from a healthy donor and found an inhibitory effect of farnesol-SUVs on TNF-α/IL-1β-stimulated IL-7 expression. Thus, the ultimate therapeutic potential of farnesol-SUVs in renal disease needs further investigation. 

## Figures and Tables

**Table 1:** Physicochemical characterization of SUV and farnesol-SUV.

	Composition	Size by Volume (nm)	Polydispersity Index
SUVs	PCsoy (25 mM)/TAP (0.5 mM)	28.2 ± 1.62	0.325
Farnesol-SUVs	PCsoy (25 mM)/TAP (0.5 mM)/Farnesol (4 mM)	31.63 ± 1.97	0.268

## Data Availability

All research data are presented in the article or in the Supplementary Materials.

## Supplementary Materials

The following supporting information can be downloaded at: https://www.mdpi.com/article/10.3390/biomedicines11123322/s1, Table S1: TNF-α/IL-1β-induced expression of inflammatory proteins in RPTECs; Table S2: TNF-α/IL-1β-induced phosphorylation of signaling proteins in RPTECs.
